# Bystanders’ thresholds for intervention in Black vs. White women’s sexual harassment

**DOI:** 10.1371/journal.pone.0296755

**Published:** 2024-02-23

**Authors:** Rebecca Schachtman, Cheryl R. Kaiser

**Affiliations:** Department of Psychology, University of Washington, Seattle, Washington, United States of America; Caleb University, NIGERIA

## Abstract

Black women’s sexual harassment is often overlooked and dismissed relative to White women’s harassment. In three pre-registered experiments, we test whether this neglect extends to bystander intervention in sexual harassment. Participants observed an ostensibly live job interview between a man manager and a Black or White woman job candidate. The manager’s questions were pre-programmed to grow increasingly harassing, and participants were asked to intervene if/when they found the interview inappropriate. A meta-analysis of the three studies (*N* = 1487), revealed that bystanders did not differ in their threshold for intervention when sexual harassment targeted the Black vs. White woman. Despite evidence for the relative neglect of Black women in responses to sexual harassment, these data suggest that bystanders may respond similarly for Black and White women.

## Introduction

The Black women and girl survivors of singer R. Kelly’s sexual abuse did not receive mass public support until nearly two years after an initial news story was published [1]. Activist Tarana Burke contrasted this delayed response with the swift response to White women survivors of producer Harvey Weinstein’s sexual harassment, saying “…we are socialized to respond to the vulnerability of white women. …[There’s a] stark difference in what it takes to get attention around Black women and girls.” [2]. The differential response to these two sexual assault scandals is consistent with scholarship showing that African American women who experience gender-based violence face greater disregard and victim-blame relative to White women from police officers, medical professionals, and other sources of support [3, 4]. This relative lack of support leaves Black women more exposed to sexual harassment and its negative psychological consequences, and unable to benefit from the buffering effects of supportive responses [4, 5]. In this work, we use an intersectional lens and experimental methods to investigate whether Black women are neglected relative to White women in one of the most immediate responses to sexual harassment- bystander intervention.

### Bystander intervention is an important response to sexual harassment

Bystanders play an important role in curbing sexual harassment, and bystanders reduce the burden on targets of harassment to respond themselves [6]. Women who confront or report their perpetrators face harmful interpersonal consequences such as being disliked or perceived as complainers [7, 8], and job-related consequences such as retaliation and backlash [9]. In light of these penalties, women do not often confront their harasser or file a report against them [10]. Although bystanders can experience retaliation from perpetrators if they intervene [11], they often face fewer consequences for confronting sexual harassment in the form of sexist comments. Relative to individuals who confront bias on their own behalf, bystanders who confront bias are perceived more positively [7, 12] and are more effective at changing perpetrators’ future behavior [13, 14]. In addition to effectively stopping harassment, bystander confrontations decrease women’s perceptions of workplace hostility and isolation and increase their identity safety and likelihood to report sexism [15–17]. At the organizational level, bystander intervention training programs for managers are associated with increases in women managers [18].

### Prototypes shape relative neglect in bystander intervention

For a bystander to determine that a woman’s treatment is sexually harassing and worthy of intervention, they must first link the target’s identity to the category “woman” [19]. Sexual harassment is strongly associated with women due to its historical legal and academic conceptualization as a form of discrimination toward women based on their gender [20, 21]. Indeed, the prototype of a sexual harassment victim largely overlaps with the prototype of a woman [22]. The category “woman” is represented by a prototype (i.e., set of features that define a category [23]); recognition of sexual harassment is facilitated when the target closely matches this prototype (i.e., is prototypical) and hindered when she deviates from it (i.e., is nonprototypical) [22]. There are two approaches to prototype fit: within-group prototypicality, which refers to variation within a group on traits, interests, and behaviors that are prototypical of that group, and between-group prototypicality, which refers to variation between intersectional groups who possess two or more subordinate social identities. More specifically, a prototypical woman is stereotypically feminine rather than masculine in her traits, interests, and behaviors (within-group prototypicality), and White rather than Black, in her intersectional identities (between-group prototypicality) [24]. In a meta-analysis of four studies, a sexual harassment target’s within-group prototypicality significantly impacted bystander intervention where bystanders had a greater threshold for intervention in a stereotypically masculine (nonprototypical) vs feminine (prototypical) woman’s harassment [25]. In the present work, we apply an intersectional lens to test if a target’s between-group prototypicality, whether the target is a Black or White woman, impacts bystander intervention in her sexual harassment.

### The costs of nonprototypicality for Black women

Black women who are sexually harassed may be neglected relative to White women because they are perceived as nonprototypical of the category “woman”. Due to their multiple subordinate identities, Black women are considered nonprototypical of both their gender and racial groups, and consequently face “intersectional invisibility” whereby their experiences are overlooked [26, 27]. For example, sexual harassment is often framed as discrimination based on gender alone, neglecting Black women’s unique experiences with sexual harassment based on their race *and* gender [21, 28]. Indeed, stereotypical attributes of the category “victim of gender discrimination”, overlap more so with stereotypical attributes of White vs Black women, and Black women are perceived as less likely targets of sexual harassment than White women [24, 29, 30].

### Black women are disproportionately impacted by sexual harassment

Although Black women are considered nonprototypical sexual harassment victims, they experience sexual harassment at higher rates of sexual harassment than White women. From 2012–2016, Black women filed three times the sexual harassment charges with the Equal Employment Opportunity Commission per 100,000 women workers as White, non-Hispanic women [31]. Additionally, progress in the last 20 years favors White women relative to Black women with reports of sexual harassment remaining unchanged for Black women but declining for White women [32]. Importantly, this disparity is not because Black women are more likely to file charges than White women. Black women face greater barriers to reporting than White women and are actually *less* likely than their White counterparts to label their experiences as sexual harassment [28]. Furthermore, Black women suffer greater psychological distress compared to White women after experiencing sexual harassment or assault [33, 34]. Despite the relative neglect of Black women in responses to sexual harassment, they are particularly common victims and are especially negatively impacted by the mistreatment.

### Bystander intervention in Black women’s sexual harassment

The relative neglect of Black women as legitimate targets of sexual harassment negatively impacts how people respond to their harassment. Indeed, empirical evidence shows that Black women are perceived as less likely gender discrimination victims (vs. White women) and, in turn, their claims are discounted to a greater extent [29]. This discounting has legal and monetary implications for Black women; an analysis of gender discrimination charges filed with the EEOC between 2011 and 2016 revealed Black (vs. White) women’s charges were more likely to receive a “no reasonable cause” determination and were awarded less money [29]. In addition to shaping responses to reports of harassment, the neglect of Black women as sexual harassment targets may impact immediate responses, like bystander intervention. In imagined sexual assault scenarios, White college women reported less intent to intervene in a Black (vs. White) woman’s assault [35]. Although bystander intervention is a promising mechanism to interrupt sexual harassment, Black women may be neglected in this response relative to White women.

### Present research

In three pre-registered experiments, we test whether bystanders have a greater threshold for intervention in a Black relative to a White woman’s sexual harassment. We manipulated a woman job candidate’s race (Black vs White) and measured participants’ points of intervention in an increasingly sexually harassing job interview between her and a man interviewer.

This work utilizes a behavioral outcome and experimental methods to examine bystander intervention. Previous research on bystander intervention often relies on self-reports of expected intervention in hypothetical scenarios. However, because people overestimate their likelihood of confronting bias [36, 37], our work uses an objective behavioral measure of bystander intervention. Additionally, in retrospective reports of bystander intervention, people may remember the most severe instance of sexual harassment in which they intervened. However, sexual harassment is most often ambiguous in nature, and by employing experimental methods, we can hold the nature of sexual harassment constant across conditions and reflective of the ways in which it manifests.

We hypothesized that bystanders would have a greater threshold for intervention in the Black relative to the White woman’s sexual harassment. We also expected participants to perceive the Black woman was sexually harassed and psychologically harmed to a lesser extent than the White woman. Finally, we hypothesized that the manager who harassed the Black (vs. White) woman would be seen as less deserving of consequences for his actions. The pre-registrations, materials, data, and code for all studies are available here: https://osf.io/pmvf8/. Scores exceeding an absolute value of three standardized residuals were winsorized to three. We found an unexpected moderation by participant gender in Study 2, so we report this exploratory analysis across all three studies for consistency. Additional exploratory analyses are presented in S1 File. All studies were approved by the University of Washington Institutional Review Board, and all participants provided written consent. The authors did not have access to identifying information during or after data collection.

## Study 1

### Methods

#### Participants

A pre-registered power analysis for a one-tailed independent samples t-test indicated 278 participants were needed for a small effect (*d* = 0.30, α = 0.05) with 80% power. To account for exclusions, we collected 350 responses in April 2022. See Table 1 for self-reported participant demographics and Table 2 for exclusions. See full pre-registration here: https://osf.io/q4pc9.

**Table 1:** Study exclusions.

				Rationale for exclusion				
Study	Initial *N*	Final *N*	Attention check ^a^	Intervention ^b^	Time ^c^	Manipulation check ^d^	Boredom ^f^	Duplicate ^g^
Study 1	350	297	18	4	1	30	0	0
Study 2	650	602	13	21	11	118 ^e^	3	0
Study 3	650	588	16	28	14	59 ^e^	1	3

^a^ Failed an attention check

^b^ Intervened after the first or second interview questions, which were not rated as sexually harassing in pre-testing, indicating they wanted to shorten their study experience.

^c^ Spent three standard deviations above the mean completion time taking the study, indicating they were distracted

^d^ Incorrectly identified the candidate’s race

^e^ Participants were included in analyses

^f^ Indicated in an open-ended response that they intervened because they were bored of the study (not pre-registered)

^g^ Duplicate ID, indicating participant took study more than once

**Table 2:** Participant demographics.

Study	Total *N*	*M* age (*SD*)	% Women	% Men	% Non-binary, Agender, or Genderfluid/queer	% Transgender	% White	% Black	% Asian	% Latinx	% Native	% Mid-Eastern	% Another
Study 1	297	40.82 (11.99)	46.46	52.19	1.01	0.34	76.77	10.44	10.10	6.06	0.34	1.01	0.67
Study 2	602	40.13 (13.03)	48.84	48.84	3.49	2.16	78.57	12.13	7.97	7.81	1.83	0.66	0.33
Study 3	588	43.97 (12.67)	57.47	40.65	0.51	0.85	82.31	8.84	6.80	5.10	1.36	0.68	0.85

*Note*. Some percentages do not add up to 100% because participants could select multiple answers for their race/ethnicity and gender. In Study 1, 0.34% of participants did not report their gender. In Study 3, 0.17% of participants used another term for their gender. Participants could only participate in one of the three studies.

#### Procedure

Following the procedure from Schachtman and colleagues [25], participants were informed that the researchers were gathering feedback for a company training their managers in a new online chat-based interviewing format. Their role was to observe a live interview and intervene if it became inappropriate. The “live” interview was, in reality, a series of ten pre-programmed increasingly harassing questions and comments from the interviewing manager directed to the job candidate with nine neutral items interspersed. See Table 3 for questions, means, and intervention frequencies.

**Table 3:** Intervention frequencies by question.

Question/statement	Sexual harassment rating	Frequencies of intervention by study		
	Pilot-testing	Study 1	Study 2	Study 3
	*M* (*SD*)	*n* (%)	*n* (%)	*n* (%)
1. First, what made you apply for this position? ^a^	1.57 (1.08)	2 (-- ^b^)	11 (-- ^b^)	11 (-- ^b^)
2. What strengths do you have as an employee? ^a^	1.43 (0.97)	2 (-- ^b^)	10 (-- ^b^)	17 (-- ^b^)
3. At this company, we really value customer satisfaction. Do you consider yourself a people pleaser? ^c^	3.00 (1.62)	88 (29.63)	71 (11.79)	89 (15.14)
4. Have you sought out any professional development opportunities in your career thus far? ^a^	--^d^	4 (1.35)	9 (1.50)	17 (2.89)
5. I’m glad you applied to this position. It’s great to see young, eager women getting involved in the business world.	3.53 (1.66)	60 (20.20)	175 (29.07)	173 (29.42)
6. If you had to describe yourself as an employee with one word, what would it be? ^a^	1.58 (0.95)	0 (0)	4 (0.66)	4 (0.68)
7. Oh before I forget, I didn’t get your phone number with your application materials. Can you give it to me so I can follow up with you? Emails seem so formal sometimes.	4.04 (1.84)	51 (17.17)	135 (22.43)	109 (18.54)
8. What do you think would be the hardest thing in this job for you? ^a^	1.66 (1.06)	2 (0.67)	1 (0.17)	0 (0)
9. In this position, you’ll have meetings with lots of older male clients. Is that something you’re up for?	4.47 (1.69)	38 (12.79)	74 (12.29)	70 (11.90)
10. Do you have any questions for me? ^a^	1.25 (0.76)	1 (0.34)	1 (0.17)	2 (0.34)
11. We work pretty late nights here. Hope your husband likes to cook!	4.74 (1.68)	18 (6.06)	38 (6.31)	49 (8.33)
12. At WorldWide, we often have to put in long hours, but the work is very rewarding. ^a^	1.57 (1.11)	1 (0.34)	0 (0)	1 (0.17)
13. Another thing I’ll add is that I personally think it’s wonderful to have a woman around the office. Hope you don’t mind having a bunch of guys around! Haha	5.02 (1.70)	12 (4.04)	36 (5.98)	33 (5.61)
14. That employee was promoted actually. So, still at WorldWide, just in a different position. ^a^	--^d^	1 (0.34)	1 (0.17)	0 (0)
15. And regarding office norms, will you be fine with wearing skirts and dresses to the office?	5.21 (1.69)	8 (2.69)	17 (2.82)	14 (2.38)
16. Any other questions? ^a^	--^d^	0 (0)	0 (0)	1 (0.17)
17. Apart from the skirts or dresses, we don’t have a strict dress code here, so it’s always fun to see what all the women in the office wear.	5.32 (1.72)	3 (1.01)	13 (2.16)	4 (0.68)
18. You know, Mary is a really nice name. Same name as my ex-wife.	5.32 (1.65)	1 (0.34)	8 (1.33)	3 (0.51)
19. Thanks for interviewing with me today, Mary. We’ll be in touch soon. ^a^	--^d^	0 (0)	0 (0)	0 (0)
Did not intervene	--	9 (3.03)	19 (3.16)	19 (3.23)
Total		297 (100)	602 (100)	588 (100)

*Note*. The pilot-testing data are the same as those reported in Schachtman and colleagues (in press). This table includes each question or statement made by the interviewer with extraneous phrasing (i.e., “Ok great,”) removed.

^a^ Neutral question/statement.

^b^ Eliminated for intervening too early and were not included in calculations.

^c^ In Study 1, this question was “Are you up for social outings with coworkers? They’re an important part of our office culture.” (*M* = 3.17, SD = 1.48).

^d^ Not pre-tested because added later as a filler item to improve interview realism and flow.

*Race manipulation*. After learning this cover story, all participants were assigned to observe an interview between Tom, the interviewing manager, and Mary, the job candidate. Participants were randomly assigned to a condition in which Mary was either a Black woman (*n* = 150) or White woman (*n* = 147). Mary’s race was conveyed via a professional headshot on her resume, created by adding professional attire and backgrounds to faces from the Chicago Face Database [38] using photo editing software. Within each condition (Black vs White), participants saw one of ten faces (cell sizes between *n* = 11–19) which were pre-matched on age, attractiveness, femininity, and masculinity. The resume content (i.e., experience, education, etc.) was identical in both conditions. Participants had to spend at least 90 seconds reviewing the resume.

*Interview observation*. Next, participants observed the “live” chat-based interview. Participants observed 19 pre-programmed exchanges between Tom and Mary via a professional messaging platform. In each exchange, Tom’s message appeared first, followed by a screen indicating Mary was typing. Next, Mary’s message appeared, followed by a screen indicating Tom was typing, and so on. Mary’s responses to Tom’s increasingly harassing comments and questions reflect those given by targets of similarly harassing interview questions in previous experimental work [39]. She used diluted language (i.e., unnecessary, or extra words and phrases), false starts (i.e., stopping mid-speech and then restarting), or repeated words to indicate slight discomfort.

Participants could intervene by indicating *Yes* or *No* after each message from Tom. They were told that intervention was anonymous, and if they intervened, the interview would stop, and the candidate would be reassigned to complete her interview with a different manager. By giving participants explicit opportunities to intervene and assuring their anonymity, we reduced common social costs that inhibit bystander intervention. We implemented this design feature to ensure sufficient variation in intervention for statistical analysis, given bystanders often fail to intervene [36, 40]. Additionally, as bystander intervention training programs gain popularity, this design reflects an increasingly prevalent workplace context in which potential bystanders receive frequent encouragement and opportunities to intervene [41].

Tom’s questions and comments became increasingly harassing as the interview progressed, which allowed us to measure participants’ thresholds for intervention (i.e., the extent to which participants allowed the harassment to escalate before intervening). Once participants intervened or watched the entire interview if they did not intervene, they responded to some self-report measures, an attention check, and a manipulation check.

In each study, we asked participants whether they found anything about their experience in the study odd. Only a small portion of participants indicated they did not believe they observed a live interview (*N* = 24 or 8.08% in Study 1, *N* = 38 or 6.31% in Study 2, and *N* = 19 or 3.24% in Study 3). These numbers did not vary by study condition (*X*^2^_S1_(1, *N* = 296) = 1.03, *p* = .311; *X*^2^_S2_(1, *N* = 601) = 1.14, *p* = .256; *X*^2^_S3_(1, *N* = 587) = 0.23, *p* = .634).

#### Measures

*Threshold for intervention*. To assess the extent to which participants allowed the harassment to escalate before intervening to stop it, we recorded the number of seconds between the beginning of the interview and when they intervened or observed the full interview using the time recording function in Qualtrics. We also used question number (1–19; non-intervention coded as 20) to measure duration to intervene. The two measures were highly correlated, *r* = 0.96, *p* < .001.

*Self-report measures*. *Perceptions of sexual harassment*. Participants responded to five items on a 7-point Likert scale (1 = *not at all*; 7 = *extremely*) rating the degree to which the candidate was sexually harassed, sexually objectified, disrespected, treated in a sexist way, and treated inappropriately during the interview, α = .91.

*Perceptions of psychological harm*. Participants completed five items on a 7-point Likert scale (1 = *not at all*; 7 = *extremely*) on the extent to which the candidate was upset, distressed, uncomfortable, traumatized, and threatened during the interview, α = .89.

*Consequences for manager*. Participants rated the extent to which the interviewing manager should be better trained on how to conduct professional interviews, should be reported, and should experience consequences on a 7-point Likert scale (1 = *strongly disagree*; 7 = *strongly agree*), α = .85.

### Results

See Table 4 for all dependent variables’ means and standard deviations.

**Table 4:** Means and standard deviations for all dependent variables by condition.

	Study 1		Study 2		Study 3	
Dependent variable	Black (*N* = 150)	White (*N* = 147)	Black (*N* = 296)	White (*N* = 306)	Black (*N* = 296)	White (*N* = 292)
	*M (SD)*	*M (SD)*	*M (SD)*	*M (SD)*	*M (SD)*	*M (SD)*
Threshold for intervention (Q#)	7.04 (4.12)	6.79 (4.18)	7.40 (3.84)	8.13 (4.42)	7.37 (3.78)	7.35 (4.04)
Threshold for intervention (seconds)	338.65 (175.24)	329.79 (175.88)	332.76 (163.37)	357.82 (183.15)	297.18 (145.73)	297.65 (152.92)
Sexual harassment	2.46 (1.32)	2.45 (1.33)	2.62 (1.32)	2.77 (1.29)	2.97 (1.46)	3.06 (1.46)
Psychological harm	2.13 (0.97)	2.10 (1.03)	2.39 (1.22)	2.52 (1.27)	2.59 (1.46)	2.71 (1.38)
Consequences for manager	4.06 (1.63)	3.97 (1.60)	4.03 (1.65) ^a^	4.17 (1.55)	-- ^b^	-- ^b^

^a^ One missing response; *N* = 295

^b^ Not collected

#### Threshold for intervention

Most participants intervened at some point during the interview (96.97%). See Table 4 for intervention frequencies by question number. Counter to our hypothesis, a one-tailed independent samples t-test revealed participants did *not* have significantly greater threshold for intervention in the Black vs White candidate’s interview by question number, *t*(295) = 0.52, *p* = .698, *d* = 0.06, 95% CI = [-0.17, 0.29] or time in seconds, *t*(295) = 0.43, *p* = .668, *d* = 0.05, 95% CI = [-0.18, 0.28].

*Gender moderation*. A pre-registered exploratory 2 (candidate race: Black vs White) x 2 (participant gender: man vs woman) ANOVA revealed participant gender did not moderate the effect by question number (*F*(1, 289) = 0.04, *p* = .843, *η*_*p*_^2^ < 0.001, 90% CI = [0, 0.01]) or by time in seconds (*F*(1, 289) = 0.002, *p* = .961, *η*_*p*_^2^ < 0.001, 90% CI = [0, 1]).

#### Self-report measures

Also counter to our hypotheses, the Black candidate was not perceived as less sexually harassed (*t*(295) = 0.09, *p* = .535, *d* = 0.01, 95% CI = [-0.22, 0.24]) or psychologically harmed (*t*(295) = 0.25, *p* = .597, *d* = 0.03, 95% CI = [-0.20, 0.26]) relative to the White candidate, and participants did not recommend fewer consequences for the Black vs White woman’s manager (*t*(295) = 0.49, *p* = .688, *d* = 0.06, 95% CI = [-0.17, 0.28]).

### Discussion

Study 1 provided no support for our hypotheses. Instead of displaying a greater threshold for intervention in Black vs White women’s sexual harassment, participants intervened around the same time for both candidates. Similarly, participants showed no significant differences by candidate race on the self-report measures. One potential explanation for the null effects is that the large quantity of individuating information on each candidate’s resumes washed out any effect of race [42]. In Study 2, we address this methodological limitation, increase the sample size, and use a sample from a different recruitment platform.

## Study 2

### Methods

#### Participants

To address the possibility of a smaller effect than originally predicted, a pre-registered power analysis for a one-tailed independent samples t-test with a small effect (*d* = 0.20, α = 0.05) and 80% power, indicated 620 participants were needed (pre-registration: https://osf.io/2grw6). To account for exclusions, we recruited 650 participants in November 2022. We recruited participants from Prolific rather than MTurk for a higher quality sample [43]. See Table 1 for exclusions and Table 2 for participant self-reported demographics.

Of note, and counter to Study 1, there was an unexpectedly high number of participants who failed to correctly identify the job candidate’s race. We suspect that the high failure rate was because participants mistakenly reported their own race rather than their perception of Mary’s race. Many participants miscategorized Mary as White in the Black condition, but as a variety of races in the White condition, and participants who miscategorized Mary as White in the Black condition, were White themselves. If our suspicions were founded, we would expect more failures in the Black vs White condition because the sample was majority White. Indeed, the exclusions varied significantly by study condition, *X*^2^(1, *N* = 118) = 12.45, *p* < .001, where more participants failed in the Black (*n* = 76) vs White (*n* = 42) condition. Given the unequal attrition, we include these 118 people who failed the manipulation check in our analyses. These exclusions do not impact the direction of our findings, and we note where they impact the significance.

#### Procedure

*Race manipulation*. As in Study 1, participants were randomly assigned to a condition in which Mary was either a Black woman (*n* = 296) or a White woman (*n* = 306). Instead of viewing the candidate’s entire resume, participants saw only Mary’s professional headshot, name, current job title, email address, and phone number. Participants in each condition saw one of five faces matched on age, attractiveness, masculinity, and femininity. Participants had to stay on the page for at least 18 seconds to ensure they viewed the manipulation.

*Interview observation*. Next, participants observed the same interview as in Study 1.

#### Measures

Threshold for intervention (by question number and by time in seconds were highly correlated, *r* = 0.97, *p* < .001), perceptions of sexual harassment (α = .91), and consequences for the manager (α = .85) were measured as in Study 1.

*Perceptions of psychological harm*. Participants completed three items on a 7-point Likert scale (1 = *not at all*; 7 = *extremely*) on the extent to which the candidate was upset, distressed, and uncomfortable during the interview, α = .89.

### Results

See Table 3 for all dependent variables’ means and standard deviations.

#### Threshold for intervention

Most participants intervened during the interview (96.84%). Counter to our hypothesis, a one-tailed independent samples t-test revealed bystanders did not have a significantly greater threshold for intervention in the Black vs. White candidate’s interview by question number, *t*(600) = -2.16, *p* = .984, *d* = -0.18, 95% CI = [-0.34; -0.02], or time in seconds *t*(600) = -1.77, *p* = .961, *d* = -0.14, 95% CI = [-0.30; 0.02]. Surprisingly, the direction of the effect indicated participants had a greater threshold for intervention in the White vs. Black candidate’s interview.

Due to the direction of the effect size, we conducted an un-preregistered two-tailed t-test, which revealed an effect in the opposite direction to our hypothesis; participants had a greater threshold for intervention in the White vs Black candidate’s interview by question number, *t*(600) = -2.16, *p* = .031, *d* = -0.18, 95% CI = [-0.34, -0.02]. The effect was only marginally significant by time in seconds, *t*(600) = -1.77, p = .077, d = -0.14, 95% CI = [-0.30; 0.02].

*Gender moderation*. A pre-registered exploratory 2 (candidate race: Black vs White) x 2 (participant gender: man vs woman) ANOVA revealed a main effect by question number opposite to what we hypothesized (*F*(1, 581) = 4.56, p = .033, *η*_*p*_^2^ = 0.01, 90% CI = [0.0003, 0.0239]); bystanders had a greater threshold for intervention in the White (*M* = 8.13, *SD* = 4.42) relative to the Black woman’s interview (*M* = 7.40, *SD* = 3.84). The main effect was qualified by a significant interaction, *F*(1, 581) = 4.04, *p* = .045, *η*_*p*_^2^ = 0.01, 90% CI = [0.0001, 0.0224]. Simple effects revealed that men participants drove the main effect with a greater threshold for intervention in the White (*M* = 9.25, *SD* = 4.87) vs Black (*M* = 7.86, *SD* = 4.11) woman’s interview, *F*(1, 581) = 8.50, *p* = .004, *η*_*p*_^2^ = 0.02, 90% CI = [0.003, 0.035]. Women did not differ significantly in their threshold for intervention when the candidate was White (*M* = 7.01, *SD* = 3.68) vs Black (*M* = 6.97, *SD* = 3.40), *F*(1, 581) = 0.01, *p* = .930, *η*_*p*_^2^ < .001, 90% CI = [0, 0.001]. When participants who failed the manipulation check were excluded, this interaction was not significant, *F*(1, 465) = 2.61, *p* = .107, *η*_*p*_^2^ = 0.01, 90% CI = [0, 0.02]. However, the omnibus main effect held, *F*(1, 465) = 5.24, *p* = .022, *η*_*p*_^2^ = 0.01, 90% CI = [0.001, 0.032].

#### Self-report measures

As in Study 1, the Black candidate was not perceived as significantly more sexually harassed (*t*(600) = -1.45, *p* = .927, *d* = -0.12, 95% CI = [-0.28, 0.04]) or psychologically harmed (*t*(600) = -1.26, *p* = .896, *d* = -0.10, 95% CI = [-0.26, 0.06]) than the White candidate, and her manager was not recommended to experience greater consequences (*t*(599) = -1.06, *p* = .855, *d* = -0.09, 95% CI = [-0.25, 0.07]).

### Discussion

As in Study 1, we did not support our hypothesis that bystanders would have a greater threshold of intervention in the Black relative to the White woman’s sexual harassment. In an exploratory analysis, we found a surprising main effect in the opposite direction to our hypotheses and an interaction with participant gender where only men participants showed the effect. However, when those who failed a manipulation check were excluded from the data, this gender moderation was not significant, and the main effect of candidate race held. Given the data quality issues and the unanticipated interaction that depended on manipulation check accuracy, we conducted a third study to further test the hypotheses. This study also strengthened the framing on the candidate race manipulation check item.

## Study 3

### Methods

#### Participants

A pre-registered power analysis for a two-tailed independent samples t-test with a small effect (*d* = 0.23, α = 0.05) with 80% power indicated 596 participants were needed. To account for exclusions, we collected 650 responses in December 2022. Participants were recruited from MTurk. See Table 1 for exclusions and Table 2 for participant demographics. See pre-registration here: https://osf.io/ehpu3.

#### Procedure

Study 3 replicated Study 2’s procedure. The manipulation check contained fewer options for the candidate’s race and reminded the participants twice that the question was about the candidate, and *not* themselves.

#### Measures

Threshold for intervention (by question number and by time in seconds were highly correlated, *r* = 0.96, *p* < .001), perceptions of sexual harassment (α = .91), and psychological harm (α = .91) were measured as in Study 1. We did not collect consequences for the manager.

### Results

#### Threshold for intervention

Most participants intervened at some point during the interview (96.77%). Consistent with Study 1 and 2, bystanders did not have a significantly greater threshold for intervention in the Black vs. White woman’s sexual harassment by question number (*t*(586) = 0.07, *p* = .473, *d* < 0.01, 95% CI = [-0.16, 0.17]), or by time in seconds (*t*(586) = -0.04, *p* = .515, *d* < 0.01, 95% CI = [-0.16, 0.16]). About half as many participants failed the manipulation check (*N* = 59) compared to Study 2, and the exclusions did not vary by study condition, *X*^2^(1, *N* = 59) = 0.05, *p* = 0.816. For consistency with Study 2 analyses, we included participants who failed in these analyses (although we pre-registered that we would exclude them). Excluding these participants (*N* = 59), produced the same direction and significance for each effect.

*Gender moderation*. As in Study 1, participant gender did not moderate the effect by question number (*F*(1, 574) = 0.80, *p* = .372, *η*_*p*_^2^ = 0.001, 90% CI = [0, 0.01]) or by time in seconds (*F*(1, 574) = 0.89, *p* = .347, *η*_*p*_^2^ = 0.002, 90% CI = [0, 0.01]).

#### Self-report measures

Again, the Black candidate was not perceived as less sexually harassed (*t*(586) = -0.74, *p* = .771, *d* = -0.06, 95% CI = [-0.22, 0.10]) or psychologically harmed (*t*(586) = -1.09, *p* = .861, *d* = -0.09, 95% CI = [-0.25, 0.07]) relative to the White candidate.

### Discussion

Similar to Study 1 and 2, Study 3 found bystanders responses to (threshold for intervention) and perceptions of (extent to which candidate was sexually harassed and psychologically harmed) sexual harassment did not neglect the Black candidate relative to the White candidate. As in Study 1, participant gender did not moderate the effect of race on threshold for intervention.

## Meta-analysis

### Threshold for intervention

We conducted a meta-analysis across the three studies (*N* = 1487) using a fixed effects approach based on Goh and colleague’s process and macro to address the discrepancies in findings for threshold for intervention. The meta-analysis revealed that, bystanders did *not* have a greater threshold for intervention in the Black vs White woman’s sexual harassment (by question number: *d* = -0.06, *SE* = .05, *Z* = -1.19, 95% CI = [-0.16, 0.04], *p* = .236; and by seconds: *d* = -0.05, *SE* = .05, *Z* = -0.95, 95% CI = [-0.15, 0.05], *p* = .342). Leveraging the statistical power of multiple studies, we found no significant effect of race on bystander intervention in sexual harassment.

### Self-report measures

The self-report measures also revealed no significant effects by candidate race in meta-analyses performed using the same method described above (sexual harassment: *d* = -0.07, *SE* = .05, *Z* = -1.34, 95% CI = [-0.17, 0.03], *p* = .180; psychological harm: *d* = -0.07, *SE* = .05, *Z* = -1.34, 95% CI = [-0.17, 0.03], *p* = .180; consequences for manager (only collected in studies 1 and 2): *d* = -0.04, *SE* = .07, *Z* = -0.60, 95% CI = [-0.17, 0.09], *p* = .594).

## General discussion

Across three experiments, we did not find evidence for the hypotheses that bystanders neglect Black relative to White women who are sexually harassed. Instead, bystanders had similar thresholds for intervention when sexual harassment targeted a Black and a White woman. The only exception to the null findings was in Study 2, where we found, opposite to our hypotheses, that bystanders had a greater threshold for intervention in the White vs. Black candidate’s interview. This pattern held only for men participants when we included all responses and for all participants when we excluded those who failed the manipulation check; however, these effects did not replicate, and the conclusions are limited by data quality. Overall, a mini-meta-analysis of the three studies found no effect of race on bystanders’ thresholds for intervention nor on any of the self-report measures (sexual harassment, psychological harm, and consequences for the manager).

Unexpectedly, these findings run counter to research showing the neglect of Black women in responses to sexual harassment. The results from Study 2 (although they did not replicate in the other studies or meta-analytically) even suggest bystanders could be more rather than less attuned to the sexual harassment of Black relative to White women. However, most prior work focuses on subsequent rather than immediate responses. For example, Black (vs. White) women’s charges with the EEOC are more likely to receive a “no reasonable cause” determination and be awarded less money [29]. Additionally, Black women receive less interpersonal support and care (e.g., more victim-blaming) from lay people, police, judges, and juries when they report or disclose their sexual assault [3, 44]. Perhaps directly witnessing sexual harassing behaviors as a bystander, rather than assessing a victim’s subsequent claim or disclosure, lessens the racial bias in how these experiences are interpreted and treated. Despite ample evidence for the neglect of Black women in responses to gender-based mistreatment, these effects did not extend to the immediate response of bystander intervention, at least as how assessed in these studies.

Another possibility is that the null results reflect aspects of our study design. Bystanders often fail to intervene, thus we attempted to create meaningful variation on this key variable by reducing the obstacles to confrontation (e.g., by allowing confrontation to be anonymous) [45, 46]. However, in doing so, we asked participants if they would like to intervene after each question or comment from the manager, which may have been too obtrusive, decreasing opportunities to observe discrimination [47]. This methodological decision follows directly from prior research with these same methods, which has indeed demonstrated that women’s gender prototypicality affects bystander intervention in sexual harassment [25]. Thus, it is unlikely that the methods alone were responsible for the null effects. Nonetheless, this design choice means we cannot generalize our findings to many situations in which bystanders face social costs for intervening. Bystanders, especially women, are less likely to intervene when the potential social costs are high vs. low [48]. As our study design removed this barrier, we may have artificially increased participants’ intervention behavior. Despite this constraint on generalizability, many organizations are in fact motivated to reduce barriers to confronting discrimination and adopt training programs that aim to create workplaces cultures that champion bystander intervention [41]. Further, with shifts toward remote work environments (e.g., Zoom, Microsoft Teams), bystander intervention can occur virtually as it did in these studies, for example through the chat function in these meetings.

Finally, despite our efforts to create a realistic online interview context, it is possible participants did not believe they were observing a real interview, which could have lowered potential social costs and inflated intervention behavior. When participants were probed to indicate whether anything about the study seemed unusual or odd, less than 10% of participants in each study indicated they knew the interview was fake. Because we did not ask them directly whether or not they believed the interview was real, it is possible that more may have been suspicious. However, we are optimistic that the question we asked, “Did you find anything about the study odd?”, encouraged disclosure of any deception awareness. This question is a modified version of a funnel debriefing procedure, which is recommended to detect awareness of deception in social psychological studies [49]. Furthermore, participants are more likely to admit their suspicions in a computer-based vs. lab-based study with an experimenter present [50].

The stimuli in our studies could also explain the null effects, as we tightly controlled for masculinity, femininity, and attractiveness of the face stimuli. In studies where these traits were not controlled, Black women are stereotyped as more masculine than White women, and this perceived masculinity leads to greater mistakes in categorizing Black women as women relative to White women [51, 52]. Additionally, Black women’s greater perceived masculinity is linked with rating them as less attractive than White women [52]. These stereotyped perceptions of Black women as more masculine and less attractive than White women may drive neglect of their sexual harassment because prototypical victims are expected to be feminine and attractive, but not masculine [24, 53]. Without variation in masculinity, femininity, and attractiveness, bystanders may have perceived the Black and White women as equally likely victims, eliminating the effect of race on their thresholds for intervention.

Finally, given the large portion of White participants in each study, our results largely describe how White bystanders respond. Black bystanders may have been more sensitive to the Black vs. White woman’s sexual harassment due to a shared social identity. We did not have sufficient power to explore this question, but it is possible that possessing a shared social identity with the victim might shape bystander action. The fact that we did not observe participant gender moderation in these studies suggests that not all shared social identities will shape bystander action. However, future research can explore this question, as well as the intersection of social identities such as gender and race on bystander action.

## Conclusion

When holding the targets’ attractiveness, femininity, masculinity, and sexual harassment constant, bystanders did not have a greater threshold for intervention in the Black vs. White woman’s sexual harassment. One interpretation of this null effect is that counter to evidence that Black women are neglected in responses to sexual harassment, and ultimately, in laws, policies, and broader feminist movements like #MeToo [20, 21, 54], the prototype bias may not shape bystander intervention. However, unlike previous research that finds race effects when features of the prototype naturally covary or are left to the perceivers’ imagination as no face stimuli are presented, our tightly controlled stimuli may have inadvertently mitigated the prototype bias. Therefore, another interpretation of the null results is that addressing the stereotypes about attractiveness, femininity, and masculinity that drive racial bias may reduce the relative neglect of Black women’s sexual harassment. Black women are particularly vulnerable to and impacted by sexual harassment, and these results offer a future direction for attenuating harmful biased responses to their harassment.

## Supporting information

S1 File Supplemental analyses. Exploratory analyses for all studies.
(PDF)
