# Supplementary material for: Bystanders’ thresholds for intervention in Black vs. White women’s sexual harassment
Source: PLoS One. 2024 Feb 23;19(2):e0296755. doi: 10.1371/journal.pone.0296755 (PMC10890732; doi:10.1371/journal.pone.0296755)
Supplement: S1 File — Exploratory analyses for all studies. (PDF) [file pone.0296755.s001.pdf]

Bystander intervention in Black vs. White women's sexual harassment

(Supplemental Analyses)

Exploratory Analyses

Gender Moderation

In all three studies, we explored whether participant gender moderated the self-report outcomes using a 2 (candidate race: Black vs. White) x 2 (participant gender: man vs. woman) ANOVA. We included only respondents who identified as a "man" or "woman" in these analyses because we did not have enough data to examine additional gender identities. We report participant gender moderation on threshold for intervention for all three studies in the main manuscript.

Sexual Harassment

Participant gender did not moderate effects on intervention on sexual harassment perceptions in any of the studies (*p*s > .275).

Psychological Harm

Participant gender did not moderate the effect of psychological harm by condition in any of the three studies (*p*s > .107).

Consequences for Manager

Participant gender did not moderate the effect of consequences for the manager by condition in Study 1 and 2 (not collected in Study 3; *p*s > .218).

External Motivation to Respond without Prejudice (EMS) Moderation

In Study 2, we explored the possibility that participants' high in external motivations to respond without prejudice may respond earlier to the Black vs White woman's sexual harassment. At the end of the study, participants responded to Plant & Devine's 1998 External Motivation to Respond without Prejudice (EMS) scale. They rated the five items on a 7-point Likert scale: 1 = *strongly disagree* to 7 = *strongly agree*: Because of today's PC (politically

correct) standards, I try to appear nonprejudiced toward Black people.; I try to hide any negative thoughts about Black people in order to avoid negative reactions from others."; "If I acted prejudiced toward Black people, I would be concerned that others would be angry with me."; "I attempt to appear nonprejudiced toward Black people in order to avoid disapproval from others."; "I try to act nonprejudiced toward Black people because of pressure from others." We predicted a significant two-way interaction between study condition and participant EMS such that participants high in EMS will intervene sooner for the Black vs White candidate, but intervention by those low in EMS will not differ significantly between conditions. The interaction was not significant (p = .886), suggesting that participant EMS did not moderate the effect of candidate race on threshold for intervention.

Controlling for Point of Intervention

In all three studies, we conducted exploratory analyses controlling for question number of intervention to address the possibility that some effects would be difficult to attain due to differing interview lengths (i.e., the later participants intervened, the more harm and harassment they would witness). We used the lm() function in the *R* package 'stats' (R Core Team, 2013) to regress each outcome of interest upon study condition and question number of intervention.

Self-Report Measures

The regression revealed no significant relationship between study condition and perceptions of sexual harassment (ps > .326), psychological harm (ps > .197), or consequences for the manger (only collected in studies 1 and 2; ps > .496) when controlling for question number of intervention.

Additional Measures

In Study 1, we explored whether participants perceived either candidate as more gender prototypical, competent, and warm. On a 7-point Likert scale from 1 = strongly agree to 7 = strongly disagree, participants rated the extent to which they believed the candidate was prototypical of her gender (feminine, masculine [reverse-coded], similar to other women, resembles the typical woman, and has a lot in common with other women, α = .80), warm (tolerant, warm, sincere, and friendly, α = .82), and competent (competent, confident, independent, and intelligent, α = .90). Each measure is analyzed using a two-tailed independent-samples t-test.

Candidate Gender Prototypicality

There was no significant difference in perceptions of the candidate's gender prototypicality when she was Black (M = 5.13, SD = 0.84) compared to White (M = 5.00, SD = 0.95), t(295) = 1.26, p = .209, d = 0.15, 95% CI = [-0.08; 0.37].

Candidate Warmth

There was no significant difference on perceptions of the Black (M = 5.44, SD = 0.87) vs. White candidate's warmth compared to White (M = 5.38, SD = 0.89), t(295) = 0.61, p = .543, d = 0.07, 95% CI = [-0.16; 0.30].

Candidate Competence

The Black candidate was perceived as marginally more competent (M = 6.00, SD = 0.80) compared to the White candidate (M = 5.81, SD = 0.87), t(295) = 1.94, p = .053, d = 0.23, 95% CI = [< 0.01, 0.45].

Mediation Analyses

In the absence of the predicted main effect in all three studies, we did not conduct any of the pre-registered mediation analyses.

Framing of Sexual Harassment

To address the possibility that people were reluctant to label sexual harassment as such, we split the sexual harassment variable into two measures in Study 2: labeled sexual harassment (the extent to which the candidate was harassed, objectified, and treated in a sexist way; $\alpha = .90$), and 2) subtle sexual harassment (the extent to which she was disrespected, offended, treated inappropriately, and treated like her boundaries were crossed; $\alpha = .90$). In both studies, two-tailed independent samples t-tests revealed no significant differences in perceptions of labeled ($t(600) = -1.63$, $p = .104$, $d = -0.13$, 95% CI = [-0.29; 0.03]) or subtle ($t(600) = -1.12$, $p = .265$, $d = -0.09$, 95% CI = [-0.25; 0.07]) sexual harassment.

References

R Core Team (2013). R: A language and environment for statistical computing. R Foundation for Statistical Computing, Vienna. http://www.R-project.org/
